# A Two Teraflop Swarm

**DOI:** 10.3389/frobt.2018.00011

**Published:** 2018-02-19

**Authors:** Simon Jones, Matthew Studley, Sabine Hauert, Alan Frank Thomas Winfield

**Affiliations:** ^1^University of Bristol, Bristol, United Kingdom; ^2^University of the West of England, Bristol, United Kingdom; ^3^Bristol Robotics Laboratory, University of the West of England, Bristol, United Kingdom

**Keywords:** swarm robotics, robot hardware, simulation, evolutionary robotics, behavior trees, distributed evolutionary algorithm, GPGPU, embodied reality modelling

## Abstract

We introduce the Xpuck swarm, a research platform with an aggregate raw processing power in excess of two teraflops. The swarm uses 16 e-puck robots augmented with custom hardware that uses the substantial CPU and GPU processing power available from modern mobile system-on-chip devices. The augmented robots, called Xpucks, have at least an order of magnitude greater performance than previous swarm robotics platforms. The platform enables new experiments that require high individual robot computation and multiple robots. Uses include online evolution or learning of swarm controllers, simulation for answering *what-if* questions about possible actions, distributed super-computing for mobile platforms, and real-world applications of swarm robotics that requires image processing, or SLAM. The teraflop swarm could also be used to explore swarming in nature by providing platforms with similar computational power as simple insects. We demonstrate the computational capability of the swarm by implementing a fast physics-based robot simulator and using this within a distributed island model evolutionary system, all hosted on the Xpucks.

## 1 Introduction

The Xpuck swarm is a new research platform with an aggregate raw processing power in excess of two teraflops, which enables new experiments that require high-individual robot computation and large numbers of robots. There are several research areas that particularly motivate the design.

Swarm robotics (Sahin, 2005) originally takes inspiration from collective phenomena in nature, including social insects, flocks of birds, and schools of fish to create collective behaviors that emerge from local interactions between robots and their environment. These swarms have the potential to be inherently robust, decentralized, and scalable. A fundamental problem of the field is the automatic design of controllers for robot swarms such that a desired collective behavior emerges (Francesca and Birattari, 2016). One common and successful approach is the use of evolutionary techniques to discover suitable controller solutions in simulated environments and the transfer of these controllers to real robots. However, this often results in lower performance due to the *reality gap* (Jakobi et al., 1995). Embodied evolutionary swarm robotics moves evolution into the swarm and directly tests controllers, avoiding the reality gap and making the swarm scalable and adaptive to the environment (Watson et al., 2002). Usually, the low-processing power of the individual robots precludes using simulation within the robots as a means of accelerating the evolutionary process. Moving computational power into the swarm would allow us to combine these approaches, the speed of evolution within simulated environments together with the adaptability of continuous reality testing.

Giving a robot the ability to answer *what-if* questions could allow a robot to evaluate courses of action or strategies in the safety of simulation, rather than in the real world where they may have potentially catastrophic consequences. The utility of this ability depends on the speed of simulation; clearly the higher the speed, the more possibilities can be tested. One use of internal *what-if* modeling is the “ethical” robot of Winfield et al. (2014), which uses simulation to allow a robot to predict the consequences of its actions or inactions on other agents and choose an ethical course of action. Another use of internal reality modeling is to detect faulty or corrupted members of a swarm by noticing deviations from predicted behavior. For safety critical applications, or where the potential consequences of actions are serious, using an unreliable communications link to remote systems would not be possible and the embodiment of the simulation within the robot is essential.

A third intriguing area where increased computational ability could be applied is in much more complex neural net controllers. Although swarm robotics as a field is inspired by social insects and other animals, the robot agents are far simpler than the organisms which inspire their creation. As a crude example, the number of neurons in an ANN controller for a swarm system rarely exceeds a dozen. Neurons in animal brains are considerably more complex and numerous; the nematode worm *C. elegans* has 302, the parasitic wasp *Megaphragma mymaripenne* has 7,400, an ant has 2.5 × 10^5^, and a honey bee has a million (White et al., 1986; Menzel and Giurfa, 2001; Polilov, 2012). The system we describe could simulate several thousand biologically plausible neurons per Xpuck.

These three areas would benefit from greatly increased processing power within the robots of a swarm, enabling either simulation of physical systems or execution of complex controllers. Many other applications of robotics such as SLAM or image processing also require high-processing power. Consumer electronics has been improving in performance for many years. Moore’s Law (Mack, 2011) observes that the number of transistors for a given cost is doubling every 18 months and their power consumption is decreasing in proportion. Over 10 years, we should expect to see a given processing performance become available with one hundredth the power consumption.^1^ This makes it now possible to build a high-computing performance swarm running on limited battery power.

In this paper, we describe the design of new swarm robotics platform that makes use of this recently available and cheap high-performance computing capability to augment the widely used e-puck robot, which many labs will already have available. We have designed it to have higher computational capability than any other swarm platforms, see Table 1, and to have a battery life at least as good as other solutions, while minimizing costs to allow the building of large swarms. We demonstrate the computational capability of the platform in two ways. First, we evaluate a fiducial tracking image processing application using the e-puck camera that would not be computationally possible on the standard e-puck. Second, and to lay the groundwork for future experiments, we implement a fast parallel physics-based robot simulator running on the GPU of the Xpuck, and use this within a distributed island-model evolutionary system to discover swarm controllers.

**Table 1:** Current and potential swarm platforms.

Product	SoC	GFLOPS (fp32)	RAM (bytes)	Price (£)
**Robot platforms**				
Kilobot	Atmel atmega328p	0.0008^a^	2 K	15
e-puck	dsPIC	0.0015^a^	8 K	650
r-one	TI Stellaris LM3S8962	0.005	64 K	165
Linux Extension Board	Atmel AT91SAM9260	0.02^a^	64 M	80^i^
Swarmbots	Intel Xscale	0.04^a^	64 M	Not known
GCTronic Gumstick	TI AM3703	1.2	512 M	600^i^
Khepera IV	TI OMAP3730	1.2	512 M	2,000
Pi-puck	Broadcom BCM2835	1.4^b^	512 M	110^i^
Pheeno	Broadcom BCM2836	7.2^c^	1 G	205
Xpuck	Samsung Exynos 5 Octa (5422)	36 + 122^d^	2 G	135
**Compute modules**				
Hardkernel XU4	Samsung Exynos 5 Octa (5422)	36 + 122^d^	2 G	70
Samsung Artik 1020	Exynos 5 Octa^e^	36 + 122^d^	2 G	98
Wandboard IMX6Q	NXP i.MX6 Quad	25^f^	2 G	120
Intrinsyc Open-Q820SOM	Qualcomm Snapdragon 820	250^g^	3 G	250
Nvidia Jetson TX1	Nvidia Tegra 210	512^h^	2 G	290

*^a^Integer only, assumes 10 integer instructions per floating point operation*.

*^b^VMLA × 0.7 GHz. VideoCore IV GPU has no OpenCL support*.

*^c^VMLA × 4 × 0.9 GHz. VideoCore IV GPU has no OpenCL support*.

*^d^CPUs A7 1.4 GHz, A15 0.8 GHz + ARM Mali-T628MP6 GPU, 4 vector multiplies, 4 vector adds, 1 scalar multiply, 1 scalar add, 1 dot product per cycle, 6 cores, each with 2 arithmetic pipelines at 600 MHz. OpenCL 1.2 full profile*.

*^e^Assumption. The product literature does not state the SoC but Samsung only used the Mali-T628MP6 in the Exynos 5 Octa family*.

*^f^Vivante GC2000 GPU only, 4 vector multiplies, 4 vector adds, 4 cores at 794 MHz, OpenCL 1.1 embedded profile*.

*^g^Very little open information, https://en.wikipedia.org/wiki/Adreno states 498.5 at 624 MHz but assumed to be fp16 rather than fp32. OpenCL 2.0*.

*^h^According to AnandTech, Ho and Smith (2015)*.

*^i^In addition to e-puck cost*.

## 2 Materials and Methods

In this section, we set out our system requirements. We outline potential computing modules. We characterize the power/performance tradeoffs of our chosen compute module and then discuss the design and implementation of the Xpuck hardware and associated system infrastructure to enable running experiments. We then detail the design and implementation of a fast physics-based robot simulator specifically tailored to the Xpuck to enable onboard evolutionary algorithms. We also describe two demonstrations of the Xpuck computational capabilities, a fiducial tracking application that could not be run on a standard e-puck, and an island model evolutionary algorithm running on multiple Xpucks.

To run experiments building on the literature, we decided that, in addition to much higher processing power, the Xpuck must meet or exceed the capabilities provided by the existing e-puck robots with additional processing boards. The e-puck is a two-wheel stepper motor-driven robot. Its sensors comprise a ring of IR proximity sensors around its periphery, a three-axis accelerometer, three microphones, and a VGA video camera. As with the Linux Extension Board (LEB), introduced by Liu and Winfield (2011), we require a battery life of at least 1.5 h and full access to the e-puck’s IR proximity and accelerometer sensors, and control of the stepper motors and LEDs. In addition, we require that the VGA camera can stream full frame at >10 fps. The Xpuck must run a full standard Linux, able to support ROS (Quigley et al., 2009). It must have WiFi connectivity. GPGPU capabilities must be made available through a standard API such as OpenCL or CUDA (Nvidia, 2007; Khronos OpenCL Working Group, 2010). We also want multicolor LED signaling capability for future visual communication experiments (Floreano et al., 2007; Mitri et al., 2009). Since many labs already have multiple e-puck robots, we wished to minimize the additional cost of the Xpuck to facilitate the construction of relatively large swarms of robots. With this in mind, we chose a target budget per Xpuck of £150.

Given the requirements, Table 1 sets out some of the current swarm platforms and potential modules that could be used to enhance the e-puck. There are a number of interesting devices, but unfortunately there are very few that are commercially available at a budget suitable to satisfy the cost requirement of £150. Within these cost constraints, of the two Samsung Exynos 5 Octa-based devices, the Hardkernel XU4 and the Samsung Artik 1020, only the XU4 was more widely available at the time of design. The Artik module became generally available in early 2017 and would be interesting for future work because of its small form-factor. There are other small form-factor low-cost modules such as the Raspberry Pi Zero, as used in the Pi-puck (Millard et al., 2017), but none that provide standard API access to GPGPU capability. For these reasons, we chose to base the Xpuck on the Hardkernel Odroid XU4 single board computer.

### 2.1 High-Performance Computing

The Hardkernel Odroid XU4 is a small single board computer based around the Samsung Exynos 5422 SoC. It has 2 GB of RAM, mass storage on microSD card, ethernet and USB interfaces, and connectors exposing many GPIO pins with multiple functions.

The SoC contains eight ARM CPU cores in a big.LITTLE^2^ formation, i.e., two clusters, one of four small low power A7 cores, and one of four high-performance A15 cores. The system concept envisages the small A7 cores being used for regular but undemanding housekeeping tasks, and the higher performing A15 cores being used when the computational requirements exceed that of the A7 cores, at the expense of greater power consumption. It also contains an ARM Mali T628-MP6 GPU, which supports OpenCL 1.2 Main Profile, allowing the relatively easy use of the GPU for GPGPU computation. Some important specifications are detailed in Table 2.

**Table 2:** Hardkernel Odroid XU4 specifications.

Spec	Details
SoC	Samsung Exynos 5 Octa (5422)
CPU organization	big.LITTLE 4 + 4
CPU big	4×ARM Cortex A15 2 GHz 4 × 32 K L1I, 4 × 32 K L1D, shared 2 M L2 25.6GFLOPS^a^
CPU little	4×ARM Cortex A7 1.4 GHz 4 × 32 K L1I, 4 × 32 K L1D, shared 512 K L2 11.2GFLOPS^b^
GPU	ARM Mali T628MP6 600 MHz 122GFLOPS^c^
Memory	2 GB LPDDR3 933 MHz PoP
Memory bandwidth	14.9 GB/s
Idle power	2 W
Maximum power	21 W

*^a^4-wide SP NEONv2 FMA × 4 × 800 MHz*.

*^b^VMLA × 4 × 1.4 GHz*.

*^c^4 vector multiply, 4 vector add, 1 scalar multiply, 1 scalar add, 1 dot product per cycle × 2 pipelines × 6 cores × 600 MHz*.

The Linux kernel supplied by Hardkernel supports full Heterogeneous MultiProcessor (HMP) scheduling across all eight cores, with the frequencies of the two clusters being varied according to the current process mix and load, the specified minimum and maximum frequencies for each cluster, and the kernel *governor* policy. ^3^ It was evident from manually changing the CPU frequencies during initial investigation that there was little subjective performance boost from using the highest frequencies, but a large increase in power consumption.

#### 2.1.1 Operating Point Tuning

Computational efficiency is an important metric, directly affecting the battery life. Initial tests showed that setting the maximum frequencies to the highest allowed by the hardware (A15—2 GHz, A7—1.4 GHz) and running a computationally heavy load caused the power consumption to exceed 15 W. To characterize the system and find an efficient operating point, we chose to perform benchmarking with a large single precision matrix multiplication using the standard BLAS API function SGEMM. This computes *C* = *αAB* + *βC*, which performs 2 *N*^2^(*N* + 1) operations for an *N* × *N* matrix. Good performance requires both high real floating point performance and good memory bandwidth. The OpenBLAS libraries (Xianyi et al., 2012) provide optimized routines capable of running on multiprocessor systems and can utilize all available processor cores. ARM provides useful application notes on implementing an efficient single precision GEMM on the GPU (Gronqvist and Lokhmotov, 2014).

Power consumption was measured for the XU4 board as a whole, using an INA231 with a 20-mΩ shunt resistor in series with the 5-V supply. A cooling fan attached to the SoC was run continuously from a separate power supply to prevent the fan control thermal regulation from affecting the power readings. Clock frequency for the A7 and A15 clusters of the Exynos 5422 were varied in 200 MHz steps from 200 MHz to 1.4 GHz for the A7, and from 200 MHz to 2 GHz for the A15 clusters, respectively. At each step, a 1,024 by 1,024 SGEMM was performed continuously and timed for at least 5 s while the power values were measured to give Floating Point Operations per second (FLOPS) and FLOPS/W. All points in the array were successfully measured except for the highest frequency in both clusters; 1.4 GHz for A7 and 2 GHz for A15, which caused the SoC temperature to exceed 95°C during the 5-s window, even with the cooling fan running, resulting in the automatic clock throttling of the system to prevent physical damage.

**Figure 1:**
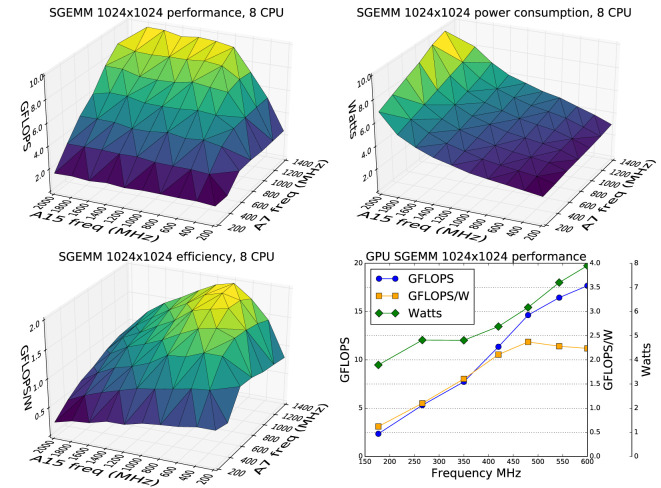
Performance, power consumption, and efficiency of the CPUs and GPU while continuously running a 1,024 × 1,024 single precision matrix multiplication. Highest efficiency for the CPU clusters is with the maximum A7 frequency of 1.4 GHz but a relatively low A15 frequency of 800 MHz. The GPU efficiency stays relatively flat above 480 MHz.

The results confirm that increasing CPU clock frequencies, particularly of the A15 cluster, produced little performance gain but much higher power consumption. Figure 1 shows that the most efficient operating point of 1.95 GFLOPS/W and 9.1 GFLOPS occurs at the maximum A7 cluster frequency of 1.4 GHz, and the relatively low A15 cluster frequency of 800 MHz. Increasing the A15 frequency to the maximum achievable of 1.8 GHz results in a 6% increase in performance to 9.7 GFLOPS but at the cost of 40% drop in efficiency to 1.21 GFLOPS/W. Because of this dramatic drop in efficiency, we fix the maximum A15 frequency to 800 MHz.

As with the CPU measurement, GPU power consumption was measured for the system as a whole, in the same way. The clock frequency of the GPU was set to each of the allowed frequencies of 177, 266, 350, 420, 480, 543, and 600 MHz and an OpenCL kernel implementing a cache efficient SGEMM was repeatedly run on both the OpenCL devices. Figure 1 shows that efficiency only declines slightly from the peak at around 480 MHz to 2.24 GFLOPS/W and 17.7 GFLOPS at the maximum 600 MHz. For this reason, we left the maximum allowed frequency of the GPU unchanged.

Note that the GFLOPS figures in these tests are much lower than the theoretical peak values in Table 2 because the SGEMM task is mostly memory bound.

### 2.2 Interface Board

**Figure 2:**
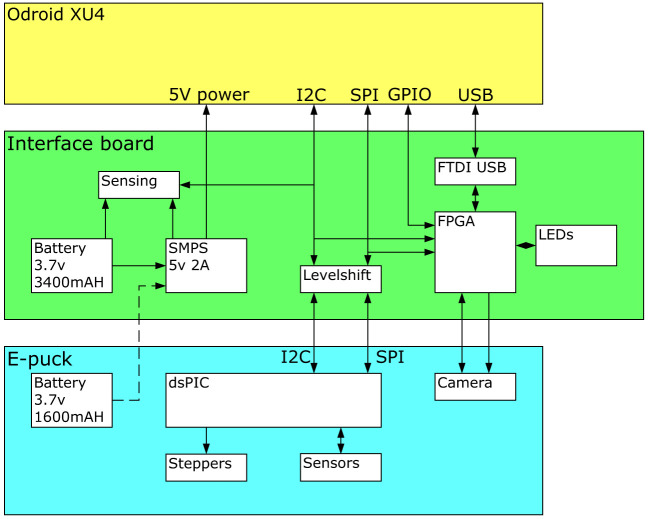
Block diagram showing the functionality of the interface board. The yellow box at the top is the XU4 single board computer, communicating over I^2^C, SPI, and USB interfaces with the interface board in green. This performs voltage level shifting, provides a USB interface to the e-puck camera, and supplies 5v power to the XU4. The e-puck in blue acts as a slave device to the XU4, running low-level proximity sensing and stepper motor control.

An interface board was created to provide power to the XU4 single board computer, interface between the XU4 and the e-puck, and provide new multicolor LED signaling. The overall structure is shown in Figure 2.

There are three interfaces to the e-puck, all exposed through the expansion connectors; a slow I^2^C bus that is used for controlling the VGA camera, a fast SPI bus that is used for exchanging data packets between the XU4 and the e-puck, over which sense and control information flow, and a parallel digital interface to the VGA camera. In each case, the interfaces have 3.3v logic levels.

The XU4 board has a 30 pin expansion connector that exposes a reasonable number of the GPIO pins of the Exynos 5422 SoC, some of which can be configured to be I^2^C and SPI interfaces. The XU4 interface logic levels are 1.8 V. A camera interface was not available, and initial investigation showed that it would not be possible to use pure GPIO pins as a parallel data input from the camera due to the high required data rate. We decided to use a USB interface to acquire camera data.

We intend to use visual signaling as a means of communication within swarms. For this purpose, we included a ring of fifteen Neopixels around the edge of the interface board. Neopixels are relatively recently available digital multicolor RGB LEDs which are controlled with a serial bitstream. They can be daisy chained in very large numbers and each primary color is controllable to 256 levels.

#### 2.2.1 Power Supply

The XU4 requires a 5-V power supply. To design the power supply, the following constraints are assumed:

- The XU4 and supporting electronics will be powered from their own battery, separate from the e-puck battery.
- The average power consumption will be 5 W.
- The peak power consumption will be 10 W.

It is immediately clear that the e-puck battery, a single-cell Li-ion type with a capacity of about 1,600 mAh, would not be able to power the XU4 as well. At a cell voltage of 3.7 V, converter efficiency of 85% and a nominal power consumption of 5 W, battery life would be at best $\frac{3.7\times 1.6\times 0.85}{5}=1$ hour, not counting the requirements of the e-puck itself. These estimates are based on battery characteristics in ideal conditions and real world values will be lower. Hence, they need for a second battery. To get a 1.5-h endurance, we assume a conservative 50% margin to account for real-world behavior, giving the requirement of $\frac{1.5\times 1.5\times 5}{3.7\times 0.85}=3.6\;Ah$.

Mobile devices are generally designed to work within a power envelope of around 5 W or the case becomes too hot to hold comfortably, see, for example, Gurrum et al. (2012). We assume that with attention to power usage, it will be possible to keep the average power at this level.

The third constraint was motivated by a survey of the readily available switch-mode power supply solutions for stepping up from 3.7 V single-cell lithium to the required 5 V. Devices tended to fall into two types—boost converters that were capable of high currents (>2 A) but with low efficiencies and large-sized inductors due to low-operating frequencies, or devices designed for mobile devices which include battery protection and have small sized inductors due to their high efficiency and operating frequency. Of the latter class, the highest output current was 2 A, with future higher current devices planned but not yet available. Measurements of the XU4 showed an idle current of 400 mA but very high current spikes, exceeding 3 A during booting. To meet the third constraint and enable the use of a high efficiency converter, the kernel was modified to boot using a low clock frequency, reducing boot current to below 1.5 A.

The power supply regulator chosen was the Texas Instruments TPS61232. It is designed for single-cell Li-ion batteries, has a very high efficiency of over 90%, a high switching frequency of 2 MHz resulting in a physically small inductor, and has battery protection with undervoltage lockout.

One aspect of the power supply design that is not immediately obvious is that the battery current is quite high, reaching 4 A as the cutoff discharge limit of 2.5 V is reached. This seriously constrains switching the input power. In fact, physically small switches capable of handling this amount of current are not readily available. For this reason, and to integrate with the e-puck, two Diodes Incorporated AP2401 high side switches were used in parallel to give electronic switching, allowing the use of the e-puck power signal to turn on the XU4 supply. The high current also necessitates careful attention to the resistance budget and undervoltage lockout settings.

To monitor battery state and energy, we use two Texas Instruments INA231 power monitoring chips, sensing across 20-mΩ resistors on the battery and XU4 side of the switching regulator. These devices perform automatic current and voltage sensing, averaging and power calculation, and are accessible over an I^2^C bus. The Hardkernel modified Linux kernel also targets the older Odroid XU3 board, which included the same power monitor chips, so the driver infrastructure is already present to access them.

**Figure 3:**
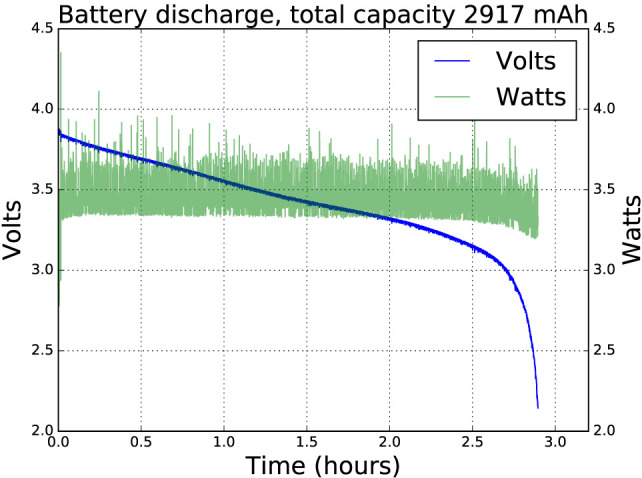
Battery life of close to 3 h while running a ROS graph with nodes retrieving camera data at 640 × 480 pixels 15 Hz, performing simple blob detection, exchanging control packets at 200 Hz with the e-puck dsPIC, and running a basic behavior tree interpreter. All the Neopixel LEDs were lit at 50% brightness and varying color, and telemetry was streamed over WiFi at an average bandwidth of 10 kB/s. The fall-off in power consumption at the 2.5-h point is due to the battery voltage falling below the threshold voltage of the blue LEDs within the Neopixels.

We used branded Panasonic NCR18650B batteries, rated at 3,400 mAh, and achieved a battery life of close to 3 h while running a ROS graph with nodes retrieving camera data at 640 × 480 pixels 15 Hz, performing simple blob detection, exchanging control packets at 200 Hz with the e-puck dsPIC and conditioning the returned sensor data, and running a simple swarm robot controller. All the LEDs were lit at 50% brightness and varying color, and telemetry was streamed over WiFi at an average bandwidth of 10 kB/s. Figure 3 shows the discharge curve. Power is relatively constant throughout at about 3.3 W except at the end, where it drops slightly. This is due to the Neopixel LEDs being supplied directly from the battery. As the voltage drops below about 3.1 V, the blue LEDs stop working, reducing the power consumption.

#### 2.2.2 Camera Interface

The e-puck VGA camera is a Pixelplus PO3030K or PO6030K, depending on the e-puck serial number. Both types have the same electrical interface, although the register interface is slightly different. It is a 640 × 480, 30 fps CMOS sensor, controlled by I^2^C, and supplies video on an eight bit parallel bus with some additional lines for H and V sync. By default, the camera provides 640 × 480 data within an 800 × 500 window in CrYCbY format. Each pixel is 16 bits and takes two clocks. The maximum clock frequency of 27 MHz gives 30 fps, with a peak bandwidth of 27 MB/s, sustained 18.4 MB/s. At our minimum desired frame rate of 10 Hz, the clock would be 9 MHz.

We considered a number of possible solutions to the problem of getting the VGA camera data into the XU4, initially focusing on implementing a USB Video Class device, which would then be simply available under the standard Linux webcam driver but available devices were relatively expensive (e.g., XMOS XS1-U8A-64 £18, Cypress Semiconductor CYUSB3014 £35, UVC app notes available for both). In the end, we settled on a more flexible approach, using the widely available and cheap FTDI FT2232 USB interface chip, together with a low power and small FPGA from Lattice.

We wanted a low-cost solution; the FT2232H is around £5, and provides a USB2.0 High Speed interface to various other protocols such as synchronous parallel, high speed serial, JTAG, etc. It is not programmable though, and cannot enumerate as a standard UVC device. The FT2232H provides a bulk transfer mode endpoint. This is not ideal for video, since it provides no latency guarantees, unlike isosynchronous mode, but since we control the whole system, we can ensure that there will be no other devices on the USB bus that could use transfer slots.

Although the FT2232H provides a synchronous parallel interface, it is not directly compatible with the camera. The FT2232H has a small amount of buffering, and uses handshaking to provide backpressure to the incoming data stream if it cannot accept new data, whereas the camera has no storage and simply streams data at the clock rate during the active 640 pixels of each line. To provide buffering and handle interfacing, we chose to use the Lattice Semiconductor iCE40HX1K FPGA. This low-cost device, less than £4 in a TQ144 package, has 96 programmable IO pins in four banks each of which that can run with 1.8, 2.5, or 3.3 V IO standards. It has 64 kB of RAM, sufficient to buffer 6.4 lines of video, or 1.3 ms at our minimum desired frame rate. We assume that the Linux USB driver at the XU4 end can handle all incoming USB data provided there is an available buffer for the data, meaning that the combined maximum latency of the user application and kernel driver must not exceed 1.3 ms to avoid underruns. Given reported sustained data rates of 25 MB/s for the FT2232H, this seems plausible, although should this not prove possible, we had the fallback position of being able to lower the camera clock frequency to a sustainable level.

The decision to use an FPGA with the large number of IOs capable of different voltage standards gave greater design freedom. There is no need for any other glue logic, and it is possible to design defensively, with a number of alternative solutions to each interface problem. It also makes possible the later addition of other peripherals. For this reason, sixteen uncommitted FPGA pins were brought out to an auxiliary connector. Lattice semiconductor provides an evaluation kit, the iCEstick, broadly similar to the proposed subsystem, allowing early development before the completion of the final PCBs.

The final system proved capable of reliably streaming camera data at 15 fps, or 9.2 MB/s, with a camera clock of 12 MHz.

#### 2.2.3 I^2^C and SPI Communications, Neopixel LEDs

All the e-puck sense and control data, except for the camera, flow over the SPI interface. It is used to control the e-puck motors and LEDs, the Neopixel LEDs on the interface board, and to read from the accelerometers and IR proximity sensors on the e-puck. The I^2^C bus is only used to set the parameters of the VGA camera.

As with the LEB, the XU4 board acts as the SPI master, providing the clock and enable signals, and the dsPIC of the e-puck the slave. SPI communication is formed of 16-bit packets. Both the master and slave have a 16-bit shift register and communication is full duplex. The master loads data into its register and signals the start of communications, followed by 16 clocks, each shifting one bit of the shift register out from the master and into the slave. Simultaneously, the slave data are shifted into the master. Between each 16 bit packet, communication pauses for long enough for the master and slave to process the received packet and prepare the next outgoing packet. This is handled in hardware with DMA at the XU4 end, but the dsPIC has no DMA and uses an interrupt routine to perform this. We used a value of 6.4 µs to ensure sufficient processing time.

The SPI signals were routed to the FPGA and the board design allows for them to be routed through it. This enables two things: first, the FPGA can watch the data from the XU4 and use fields within that to control its own peripherals, currently the Neopixel LEDs, second, it allows the insertion of data into, or the modification of the return messages from the e-puck.

The FPGA contains additional logic to interpret fields within the SPI packet for controlling the Neopixel LEDs. These data are stored in a buffer within the FPGA and used to generate the appropriately formatted serial stream to the LEDs.

### 2.3 Physical Design

**Figure 4:**
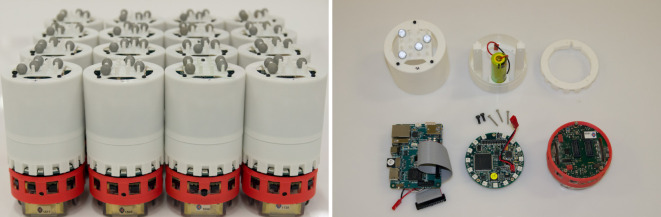
Left: 16 assembled Xpucks. Right: major components, left to right, top to bottom. Outer 3D printed shell showing Vicon tracking reflectors in unique pattern on top. Support chassis, which holds the XU4 single board computer and the LiION battery. Spacer ring, locating the chassis above the PCB and reflecting the upward facing LEDs outwards. XU4 computer, with leads for power and data. Interface PCB. Base e-puck, with red 3D printed skirt.

**Figure 5:**
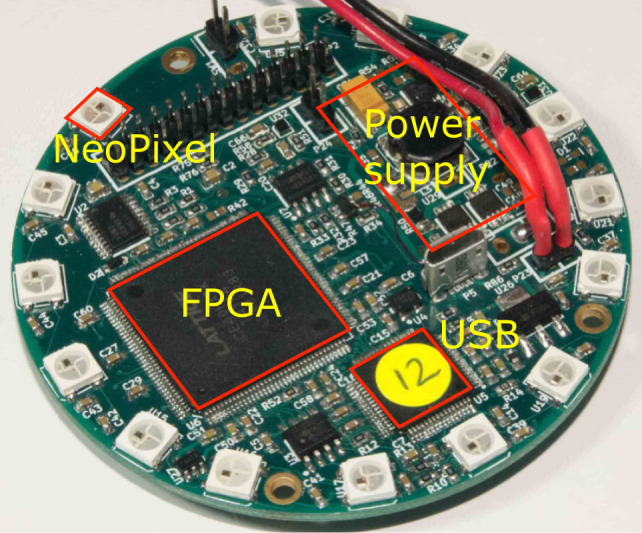
Interface board PCB, showing the boost converter PSU for the XU4 5v supply, the FPGA and USB interface, the VGA camera and SPI level shifting, and the 15 Neopixels.

The interface board is 70 mm in diameter, the same as an e-puck. It sits on top of the base e-puck. Above this, the XU4 board is held vertically within a 75-mm diameter cylindrical 3D printed shell, which also holds the battery. Flying leads from the XU4 for the GPIO parallel and the USB interfaces, and for the power supply, connect to the interface board. Figure 4 shows 16 completed Xpucks, and the major components of the assembly. Figure 5 shows details of a populated interface board.

### 2.4 Software and Infrastructure

The swarm operates within an infrastructure that provides tracking, virtual sensing, and message services. To facilitate this, the Xpucks run a full featured distribution of Linux and ROS, the Robot Operating System (Quigley et al., 2009). This gives access to much existing work: standard libraries, toolchains, and already existing robot software. Given the close dependence of ROS on Ubuntu we chose to use Ubuntu 14.04.4 LTS, running ROS Indigo.

#### 2.4.1 Real-time Kernel

The standard Linux kernel is not hard real-time, i.e., it does not offer bounded guarantees of maximum latency in response to events. One of the tasks that are running on the XU4 that requires real-time performance is the low-level control loop comprising the SPI data message exchange with the e-puck. The maximum speed of the e-puck is about 130 mm/s. A distance of 5-mm corresponds to about 40 ms. It would be desirable to have a control loop with a period several times faster than that, one commonly used in e-puck experiments is 100 Hz, or *t_control_* = 10 ms. The minimum time for the control loop to respond to a proximity sensor is two SPI message lengths, so to achieve a 10-ms control period, we need an SPI message period *t_period_* < 5 ms. Assuming a 5-MHz SPI clock with a message comprising 32 16-bit packets and a 6.4-µs interpacket gap, the total time per message is *t_message_* = 307 µs. This gives a budget of *t_period_* − *t_message_* = 4.7 ms for processing and latency. Measurements using cyclictest ^4^ over 500,000 loops of 1 ms, or about 8 min, with the *Server* preemption policy kernel while running SPI message exchange at 200 Hz showed figures of 13.9 ms, and even when running the *Low-Latency Desktop* preemption policy this was above 3.5 ms. This leaves little margin for processing.

We used the PREEMPT-RT patch (Rostedt and Hart, 2007), which modifies the kernel to turn it into a real-time operating system (RTOS), able to provide bounded maximum latencies to high priority real-time user tasks. With the RTOS kernel, the measured latencies while running SPI message exchange never exceeded 457 µs over several hours running at 200 Hz.

#### 2.4.2 Resilient Filesystem

One of the important issues when making reliable Linux embedded systems is how to deal with unexpected power removal. Linux filesystems, in general, are likely to be corrupted if the power is removed while they are performing a write. Even journaling filesystems like ext4 are prone to this. This is why Linux needs to be properly shut down before power is removed, but this is simply not practical for an experimental battery-powered system. Disorderly shutdowns will happen, so this needs to be planned for.

We implement a fully redundant filesystem with error checking using BTRFS (Rodeh et al., 2013) as described in a StackExchange answer. ^5^ BTRFS is modern journaling filesystem that supports on-the-fly compression and RAID and is capable of self-healing, provided there are redundant copies of the data. The idea is that we create two partitions on the same SD card and mount them as a completely redundant RAID1 array. Any filesystem corruption will be seen as a mismatch between checksum and file, and the redundant copy on the other partition used to replace the corrupt version. This has proven to be very reliable so far, with no corrupted SD cards.

#### 2.4.3 Arena Integration

**Figure 6:**
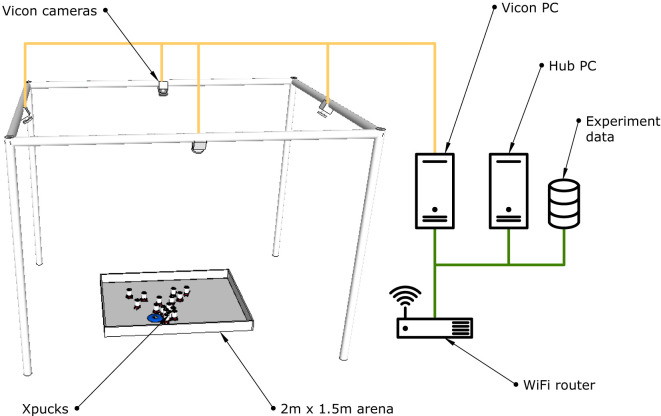
The Xpuck arena. Experiments take place within a 2 m × 1.5 m area surrounded by walls slightly higher than the height of the Xpucks. Each Xpuck has a unique pattern of spherical reflectors on their top surface to enable the Vicon motion tracking system to identify each individuals pose. The Vicon PC is dedicated to managing the Vicon system and makes available a stream of pose data. The Hub PC is responsible for all experiment management, data logging, and virtual sense synthesis.

The Xpucks work within an arena which provides the infrastructure for experiment control, implementing virtual senses if needed, and for logging, see Figure 6. It is area 2 m by 1.5 m equipped with a Vicon tracking system and an overhead network webcam. Each Xpuck has a USB WiFi dongle, and the arena has a dedicated WiFi access point. For robustness, each Xpuck has a fixed IP address, and the standard scripts are replaced with a script that continually checks for connectivity to the access point and attempts reconnection if necessary.

Software called the *switchboard* runs on the *Hub* server and is responsible for the distribution of experiments to the Xpucks, their initiation, and the logging of all experiment data. Each Xpuck automatically starts a ROS node at boot which connects to the Hub over ZeroMQ sockets (Hintjens, 2013) supplying a stream of telemetry about the physical state of the Xpuck, including battery levels and power consumption, temperature, sensor values, and actuator settings. The switchboard sends timestamps, virtual sense data, and can command the ROS node to download and execute arbitrary experiment scripts, which would typically set up a more complex ROS graph for the robot controller, which in turn will run the experiment upon a trigger from the switchboard. Controllers are always run locally on the Xpucks. This is all controlled either from the command line on the Hub or with a GUI giving visibility to important telemetry from the swarm.

Each Xpuck is marked with a unique pattern of reflectors recognized by the Vicon system. There are four reflectors arranged on a 4 × 4 grid with spacing of 10 mm. We used a brute force search to find unique patterns for each of the 16 Xpucks. Because of the size of the marker pattern and of the Xpucks themselves, there should be no ambiguous conditions when Xpucks are close to each other. This has proved effective with unambiguous detection even when all 16 Xpucks were placed packed together in the arena.

The switchboard software connects to the Vicon system and receives pose messages at the update rate of 50 Hz. This is used to log the absolute positions of the Xpucks during experiments and also to synthesize virtual senses included in the outgoing streams of data from the switchboard to the Xpucks. Range and bearing is an important sense in swarm robotics experiments, which we can construct directly using the e-pucks IR proximity sensors or with additional hardware (Gutiérrez et al., 2009a,b). We can also synthesize range and bearing information from the Vicon data with behavior defined by a *message distribution model*, which allows us to specify parameters such as range, noise, and directionality. There is the capability for Xpucks to send broadcast messages consisting of their ID, this is disseminated by the switchboard according to the message distribution model. Messages received have no content, but are an indication that the sender and the receiver can communicate, actual data transfer can take place point-to-point. In this, we take inspiration from O’Dowd et al. (2014), who use IR communication between e-pucks to establish if contact is possible, data transfer then taking place over WiFi.

### 2.5 GPGPU Robot Simulator

In this section, we describe the design and realization of a fast parallel physics-based 2D multi robot simulator running on the Xpuck SoC GPU.

To perform onboard evolution of controllers or to evaluate multiple what-if scenarios, we need to be able to run many simulations much faster than real-time. A typical evolutionary algorithm might have a population of *p* potential solutions. Each of these needs to be evaluated for fitness by running *r* simulations with different starting conditions. Many generations *g* of evaluation, selection, combination, and mutation take place to produce fitter individuals. Typically, *p*, *r*, *g* might be (50, 10, 100). One scenario we envisage is evolving a controller for the next fixed interval Δ*t* of real time. During the current time interval, we need to complete *n*_*sims*_ = *prg* simulations of that time Δ*t*, or:

(1) $$\frac{n_{\mathrm{sims}}\cdot t_{\mathrm{real}}}{t_{\mathrm{sim}}}<1$$

where *t_sim_* is the simulated time and *t_real_* is the wall clock time for that simulated time. It is generally the case (Vaughan, 2008; Jones et al., 2015) that multi robot simulation time is proportional to the number of agents being simulated. We define a simulator speed using the robot acceleration factor:

(2) $$r_{\mathrm{acc}}=\frac{n_{\mathrm{robots}}\cdot t_{\mathrm{sim}}}{t_{\mathrm{real}}}$$

where *n_robots_* is the number of robots, *t_sim_* and *t_real_* as above. With equation (1) we get a required *r_acc_* of:

(3) $$r_{\mathrm{acc}}>n_{\mathrm{sims}}\cdot n_{\mathrm{robots}}.$$

We can see that if we are using a single simulator, the required *r*_*acc*_ increases with the number of robots being simulated. But if we run a distributed evolutionary algorithm and have a simulator embodied in each robot, the required *r*_*acc*_ simply becomes:

(4) $$r_{\mathrm{acc}}>n_{\mathrm{sims}}.$$

For the example above, we therefore require a simulator with *r*_*acc*_ > 50,000.

There is a basic trade-off between simulator fidelity and speed. Typical values of *r*_acc_ when running on a PC are 25 for a full 3D physics simulation like Gazebo, 1,000–2,000 for 2D ^6^ arbitrary shape simulators with relatively detailed sensory modeling like Stage (Vaughan, 2008), and ARGoS (Pinciroli et al., 2011), and 50,000–100,000 ^7^ for constrained geometry 2D physics game engines like Box2D (Catto, 2009). There is also a cost to generality; the critical path in stage is the ray-tracing operation for modeling of distance sensors, necessary to handle arbitrary object shapes in the simulated world. We show in Jones et al. (2016) that a constrained geometry 2D physics engine simulator is capable of being used to evolve swarm controllers which transfer effectively to the real world, so this motivates our simulator design.

To get good performance on an application running on a GPU, it is necessary that there is a large number of work items that can be performed in parallel. The Mali Midgard GPU architecture present in the Exynos 5422 SoC of the XU4 has six shader cores, each of which can run 256 threads simultaneously. To keep the cores busy, it is recommended that a kernel be executed over hundreds or thousands of work items, depending on its resource usage. We therefore need to design our simulator to have parallelism at least in the hundreds to take advantage of the GPU and be sufficiently constrained in scope that we avoid the costs of generality; by using only straight lines and circles in our simulation, collisions and sensor intersections can be calculated cheaply by geometry, rather than expensive ray-tracing.

#### 2.5.1 Simulation Model

The simulation models up to 16 Xpuck robots within a 2 m × 1.5 m rectangular arena centered about the origin, with the edges of the arena surrounded with immovable walls. As well as the Xpuck robots, there can be other inert round objects that can be pushed by the Xpucks. The reference model for the robots is given in Table 3, this describes the sensors and actuators that are exposed to the robot controller.

**Table 3:** Robot reference model.

Input variables	Values	escription
*P*_*i*∈{1,2,…,8}_	[0,1]	Proximity sensor *i*, 0 = nothing in range, 1 = object adjacent to sensor
*θ*	[−*π, π*)	Compass, giving pose angle in world frame
n∈N	{0, …, 16}	Number of neighboring Xpucks
(*r*, ∠*b*)_*i*∈{1, …, *n*},*n* ≠ 0_	([*r_min_*, *r_max_*], [−*π, π*))	Range and bearing of neighbor *m*
*R*_*i*∈{*left,center,right*}_	{0, 1}	Red blob detection
*G*_*i*∈{*left,center,right*}_	{0, 1}	Green blob detection
*B*_*i*∈{*left,center,right*}_	{0, 1}	Blue blob detection

**Output variables**		

*v*_*i*∈{*left,center,right*}_	[−*v_max_*, *v_max_*]	Left and right wheel velocities

**Constants**		

*t_update_*	100 ms	Controller update period
*r_min_*	0.075 m	Minimum range and bearing range (center to center)
*r_max_*	0.5 m	Maximum range and bearing range (center to center)
*v_max_*	0.13 ms^−1^	Maximum wheel velocity
∠*q_i_*_∈{1,2,…,8}_	0.297, 0.855, 1.571, 2.618, −2.618, −1.571, −0.855, −0.297	Angle of proximity sensor *i*
*p*_max_	0.04 m	Proximity sensor maximum range

We can divide the simulation into three sections; *physics*, *sensing*, and *control*. *Physics* handles the actual physical behavior of the robots within the arena, modeling the dynamics of motion, and collisions in a realistic way. *Sensing* constructs the input variables described in the robot reference model from the locations and attributes of the objects within the simulated world. *Control* runs a robot controller for each simulated robot, responding to the reference model inputs, and producing new output variables, resulting in the robot acting within the world.

There are three types of object within the world: the arena walls, the Xpucks, and inert objects. The walls are immoveable and are positioned horizontally and vertically symmetrically about the origin. Xpucks, which are round and colored, can sense each other with their camera, proximity sensors and range and bearing, and can move with two-wheel kinematics. Inert objects, which are round and colored, can be sensed by Xpuck cameras but not by the proximity sensors because they are low in height. They move only due to collisions.

##### 2.5.1.1 Physics

The physics core of the simulation is based on work by Gaul (2012). There are only circular bodies, which are rigid and have finite mass, and the walls, which have infinite mass. Interactions between bodies are governed by global attributes of coefficients of static and dynamic friction, and restitution. Interactions between the bodies and the arena floor are governed by individual attributes of mass and coefficient of friction. The physical state of each body *i* is described by the tuple $S_{i}\left(\text{x},\text{v},\theta ,\omega \right)$ representing position, velocity, angle, and angular velocity.

The equations of motion governing the system are $\dot{\text{v}}\;=\;\frac{1}{m}\text{F},\dot{\omega }\;=\;\frac{1}{I}\tau ,\dot{\text{x}}\;=\;\text{v},\dot{\theta }\;=\;\omega$. They are integrated using the symplectic Euler method (Niiranen, 1999) which has the same computational cost as explicit Euler but better stability and energy preserving properties.

Collisions between bodies are resolved using impulses. For each pair of intersecting bodies, a contact normal and relative velocity are calculated, producing an impulse vector which is used to instantaneously change the linear and angular velocities of the two bodies. This is iteratively applied multiple times to ensure that momentum is transferred in a physically realistic way between multiple contacting bodies.

Collision detection between pairs of bodies with a naive algorithm is *O*(*n*^2^) so most physics simulators handling a large number of bodies (100 s upwards) use a two stage process with a *broadphase* step that eliminates a high proportion of pairs that cannot possibly be in collision, before the *narrowphase* step that detects and handles those bodies that are actually colliding. But we have only a maximum of 21 bodies (4 walls, 16 robots, 1 object) which means that any broadphase step must be very cheap to actually gain performance overall. We tried several approaches before settling on a simple binning algorithm: each object is binned according to its *x* coordinate, with bins just larger than the size of the objects. A bin contains a bitmap of all the objects within it. Objects can only be in collision if they are in the same or adjacent bins so the or-combined bitmap of each two adjacent bins is then used to form pairs for detailed collision detection.

The two-wheel kinematics of the robots are modeled by considering the friction forces on each wheel due to its relative velocity to the arena surface caused by the wheel velocity and the object velocity. Friction force is calculated as Coulomb but with *μ* reduced when the velocity is close to zero using the formulation in Williams et al. (2002): $\mu =\mu_{\max}\frac{2\cdot \arctan\left(k*v\right)}{\pi }$. With the same justification as Williams et al. (2002), we chose *k* = 20 empirically to ensure numerical stability. The forces on each body are resolved to a single force vector ***F*** and torque *τ*. Non-robot objects simply have zero wheel velocities.

The noise model is a simplified version of that described by Thrun et al. (2005). Three coefficients, *α*_1_, *α*_2_, *α*_3_, control, respectively, velocity-dependent position noise, angular velocity-dependent angle noise, and velocity-dependent angle noise. So position and angle are modified: $\text{x}\prime =\text{x}+\text{v}\cdot s\left(\alpha_{1}\right),\theta \prime =\theta +\omega \cdot s\left(\alpha_{2}\right)+|\text{v}|\cdot s\left(\alpha_{3}\right)$ where *s*(*σ*) is a sample from a Gaussian distribution with SD *σ* and mean of zero. Because the noise model is on the critical path of position update and the calculation of even approximate Gaussian noise is expensive, we use a pre-calculated table of random values with the correct distribution.

The physics integration timestep is set at 25 ms for an update rate of 40 Hz. This value was chosen as a trade-off performance and physical accuracy, giving 4 physics steps per controller update cycle.

##### 2.5.1.2 Sensing

There are three types of sensors that need to be modeled. Each Xpuck has eight IR proximity sensors arranged around the body at a height of about 25 mm. These can sense objects out to about 40 mm from the body. The reference model specifies that the reading varies from 0 when nothing is in range, to 1 when there is an object adjacent to the sensor. Similar to the collision detection above, the maximum sensor range is used to set the radius of a circle about the robot which is tested for intersection with other objects. For all cases where there is a possible intersection, a ray is projected from the robot at each sensor angle and a geometrical approximation used to determine the location of intersection with the intersected body and hence the range. This process is actually more computationally expensive than collision detection, but only needs to take place at the controller update rate of 10 Hz.

The second and third types of sensor are the camera blob detection and the range and bearing sense. Blob detection splits the camera field of view into three vertical segments and within each segment, detects the presence of blobs of the primary colors. Range and bearing sense counts the number of robots within 0.5 m and produces a vector pointing to the nearest concentration. Together they are the most computationally expensive of the senses to model. They necessarily traverse the same data structures and so are calculated together.

To model the camera view, we need to describe the field of view subtended by each object, what color it is, and whether it is obscured by nearer objects. We implement this by dividing the visual field into 15 segments and implementing a simple *z*-buffer. Each object is checked and a left and right extent derived by geometry. The segments that are covered by these extents have the color of the object rendered into them, if the distance to the object is less than that in the corresponding *z*-buffer entry. As each object is checked, the distance is used to determine if the range and bearing information needs to be updated.

In the real robot arena, range and bearing is implemented as virtual sensing using a Vicon system and communication over WiFi. There is significant latency of around 100–200 ms between a physical position and an updated range and bearing count and vector reaching the real robot controller. Also, the camera on each Xpuck has processing latency of a similar order. For this reason and due to the computational cost, this sensor information is updated at half the controller rate, or 5 Hz.

##### 2.5.1.3 Controller

The controller architecture we use is behavior tree based (Champandard, 2007; Ogren, 2012; Colledanchise and Ogren, 2014; Scheper et al., 2015; Jones et al., 2016). Originating in the games industry for controlling non-player characters, behavior trees are interesting for robotics because they are hierarchical, allowing encapsulation and reuse of sub-behaviors, human readable, aiding analysis of evolved controllers for insight, and amenable to formal analysis. A behavior tree consists of a tree of nodes and a *blackboard* of variables which comprise the interface between the controller and the robot. At every controller update cycle, the tree of each robot is evaluated, with sensory inputs resulting in actuation outputs. Evaluation consists of a depth-first traversal of the tree until certain conditions are met. Each agent has its own blackboard and state memory, the tree is shared by all agents running the same controller. In our case, we are running homogeneous swarms, so within a particular simulation, only one tree type is used, with each simulated robot running its own instance.

##### 2.5.1.4 Implementation of Simulator on GPU

To best exploit the available performance of the GPU, our implementation must have a high degree of parallelism. We achieve this by running multiple parallel simulations almost entirely within the GPU. The limit to parallelization of running multiple simulations for an evolutionary algorithm is the number of simulations per generation; it is necessary to completely evaluate the fitness of the current generation to create the individuals that will make up the next generation. With the numbers given above, this would be 500 simulations, below what would normally be recommended to keep the GPU busy, but long-lasting threads ensure the GPU is fully utilized.

As we implemented the simulator, it actually turned out that memory organization was the most critical element for performance. Each of the four cores within the first core group of the GPU ^8^ has a 16-kB L1 data cache and a 256 L2 cache shared between them. Ensuring that data structures for each agent were minimized, and that they fitted within and were aligned to a cache line boundary resulted in large performance improvements. Memory barriers between different stages of the simulation update cycle ensured that data within the caches remained coherent and reduced thrashing. As performance improved and the memory footprint changed, the effect of workgroup size and number of parallel simulations was regularly checked. We used the DS-5 Streamline ^9^ tool from ARM to visualize the performance counters of the GPU which showed clearly the memory-bound nature of the execution. Profiling of OpenCL applications is difficult at anything finer than the kernel level, so there was much experimentation and whole application benchmarking.

### 2.6 Image Processing Demonstration

The high computational capability of the Xpuck makes it possible to run camera image processing algorithms not possible on the e-puck on its own or enhanced with the Linux Extension Board. To demonstrate this and to evaluate the performance of the camera, we implement ArUco marker tracking (Garrido-Jurado et al., 2014) and test it with the onboard camera. ArUco is a widely used library that can recognize square black and white fiducial markers in an image and generate camera pose estimations from them. In this demonstration, we use the marker recognition part of the library and test the tracking under different distances and Xpuck rotational velocities.

A ROS node was written to apply the ArUco ^10^ marker detection library function to the camera image stream and to output the detected ID and pixel coordinates on a ROS topic. Default detection options were used and no particular attention was paid to optimization.

Two experiments were conducted. In both cases, we used video from the Xpuck camera at a resolution of 320 × 240 and a frame rate of 15 Hz. First, we measured the time taken to process an image with the detection function under conditions of no markers, four 100 mm markers in a 2 × 2 grid, and 81 20 mm markers in a 9 × 9 grid. Frame times were captured for 60 s.

Second, we affixed four ArUco tags of size 100 mm with different IDs to locations along the arena walls. An Xpuck was placed in three different locations within the arena and commanded to rotate at various speeds up to 0.7 rad/s. Data were collected for 31,500 frames. Commanded rotational velocity, Vicon tracking data, and marker tracking data were all captured for analysis.

The data were analyzed in the following way: each video frame is an observation, which may have markers present within it. Using a simple geometrical model, we predict from the Vicon data and the known marker positions whether a marker should be visible in a given frame and check this against the output of the detector for that frame. From this, we derive detection probability curves for different rotation speeds.

### 2.7 In-Swarm Evolution Demonstration

One of our motivations for moving computation into the swarm is to tackle the scalability of swarm controller evolution. To demonstrate both the computational capability of the Xpuck swarm and scalability, we implement an island model evolutionary algorithm and demonstrate performance improvement when running on multiple Xpuck robots.

The island model of evolutionary algorithms divides the population of individuals into multiple subpopulations, each of which follows its own evolutionary trajectory, with the addition of *migration*, where some individuals of the subpopulations are shared or exchanged with other subpopulations. Island model evolutionary algorithms enable coarse-grained parallelism, with each island corresponding to a different compute node, and sometimes outperform single population algorithms by maintaining diversity (Whitley et al., 1999). Even without that factor, the ability to scale the size of total population with the number of compute nodes hosting subpopulations is desirable for a swarm of robots running embodied evolution.

#### 2.7.1 Implementation of Island Model

On each Xpuck, we run a genetic algorithm evolving a population of behavior tree controllers similar to that described in Jones et al. (2016) using methods from Genetic Programming (Koza, 1992). The parameters are described in Table 5. Evolution proceeds as follows: an initial subpopulation of *n_sub_* individuals is generated using the Koza’s *ramped_half_and_half* procedure, detailed in Poli et al. (2008), with a depth of *n_depth_*. Each individual is evaluated for fitness by running *n_sims_* simulations with different starting conditions and averaging the individual fitnesses. The subpopulation is sorted and the top *n_elite_* individuals are copied unchanged into the new subpopulation. The remaining slots are filled by tournament selection of two individuals with replacement followed by a tree crossover operation, with random node selection biased to internal nodes 90% of the time (Koza, 1992), to create a new individual. Then, every parameter within that individual is mutated with probability *p_mparam_*, followed by mutating every node to another of the same arity with probability *p_mpoint_*, followed by replacing a subtree with a new random subtree with probability *p_msubtree_*. This new population is then used for the next round of fitness measurement.

The genetic algorithm is extended to the island model in the following way: after every *n_epoch_* generations, each Xpuck sends a copy of the fittest individual in its subpopulation to its neighbors. They replace the weakest individuals in their subpopulations. Currently, this is mediated through a *genepool server*, running on the Hub PC, although direct exchange of genetic material between individual Xpucks is also possible using local IR communication. This server maintains the topology and policy for connecting the islands. This may be physically based, drawing on the position information from the Vicon. It is important to note that server provides a way to abstract and virtualize the migration of individuals; in the same way, we use the Vicon information to provide virtual sensing. When the server receives an individual from a node, it replies with a set of individuals, according to the policy. These are used to replace the least fit individuals on the requesting node. The process is asynchronous, not requiring that the nodes execute generations in lockstep. The policy for this experiment is to make a copy of each individual available to every other node, so with *n_nodes_* nodes the migration rate is $r_{\mathrm{migration}}=\frac{n_{\mathrm{nodes}}-1}{n_{\mathrm{sub}}\;\cdot \;n_{\mathrm{epoch}}}$.

#### 2.7.2 Task and Fitness Function

We evolve a behavior tree controller for a collective object movement task. The task takes place in a 2 m × 1.5 m arena with the origin at the center and surrounded by walls greater than the height of the Xpucks. The walls and floor are white. A blue plastic frisbee of 220 mm diameter is placed at the origin. Nine Xpucks with red skirts are placed in a grid with spacing 100 mm centered at (−0.8, 0) and facing rightwards. The goal is to push the frisbee to the left. Fitness is based on how far to the left the frisbee is after a fixed time. An individual Xpuck can push the frisbee, but only at about half the full Xpuck speed, so collective solutions have the potential to be faster. The swarm is allowed to execute its controller for 30 s. After this time, the fitness is given by equation (5).

(5) $$f=\left\{\begin{array}{cc} r_{\mathrm{derate}}\frac{-x}{1-l_{\mathrm{frisbee}\text{\_}\mathrm{radius}}}, & \text{for}\;\text{x}\;<\;0\\ 0,\; & \text{otherwise} \end{array}\right.$$

where *x* is the x-coordinate of the center of the frisbee, and *r_derate_* is a means of bloat control, proportionately reducing the fitness of behavior trees which use more than 50% of the resources available. To show scalability with increasing numbers of Xpucks, we compare two scenarios, first a single Xpuck running a standalone evolution and second six Xpucks running an island model evolution. In both cases, the parameters are as in Table 5. With the island model, every *n_epoch_* = 2 generations, a node sends to all its neighbors a copy of its fittest individual and receives their fittest individuals, using these to replace its five least fit individuals, giving a migration rate *r_migration_* = 0.078. Each scenario is run ten times with different initial random seeds.

## 3 Results

### 3.1 Xpucks

The total cost of 25 Xpucks was £3,325, or £133 each. This includes all parts, PCBs, XU4 single board computers, and batteries. It does not include assembly or the base e-pucks, which cost around £700. Although it should be possible for a university technician to assemble the boards in small quantities, the approximate costs per board for factory PCB assembly were £17 for 25 boards, dropping rapidly to £6 for 100 boards. ^11^ It is our intention to make the design open source and freely available. ^12^

Currently, we have 16 assembled and functional robots. Battery life when running a moderate computational load is close to 3 h. When continuously running the extremely computationally demanding evolutionary algorithm described, the battery life dropped to around 1 h 20 min.

### 3.2 Simulator

Table 4 shows the results of running parallel simulations for a simulated time of 30 s. Each simulation consists of 16 robots running a simple controller for exploration with basic collision avoidance, and one additional object that can be pushed by the robots. The effect of running different numbers of parallel scenes and with various different levels of functionality enabled is shown. *t_rss_* is the time to simulate one robot second. $t_{\mathrm{rss}}=\frac{1}{r_{\mathrm{acc}}}$, so the required acceleration factor of 50,000 corresponds to *t_rss_* = 20 *μ*s. It can be seen that the requirement is met when running 256 simulations in parallel, with *t_rss_* = 17 *μ*s. It is interesting to note that when running 512 simulations, the performance is better with all functionalities except the controller enabled. We surmise that, when running the controller, the total working set is such that there is increased cache thrashing with 512 parallel simulations.

**Table 4:** Speed of simulator with various functionalities enabled.

	256 simulations			512 simulations		
Functionality	*t_rss_*	Δ*t_rss_*	%	*t_rss_*	Δ*t_rss_*	%
Physics	6.9	6.9	40	6.7	6.7	31
+Sensors	11	4.5	26	11	4.0	19
+Camera and R&B	15	3.1	18	14	3.3	16
+Controller (all functionality)	17	2.6	16	21	7.2	34

*16 robots, 1 passive object, basic exploration and collision avoidance controller. Tested over five runs with 256 and 512 parallel simulations. t_rss_ is time (μs) per robot simulated second. With 256 parallel simulations, the physics functionality dominates at 40% of the processing time, but with 512 parallel simulations, controller processing is the largest proportion*.

The performance of the simulator running on the Xpuck GPU is comparable to the same code running on the CPU of a much more powerful desktop system and at least ten times faster than more general purpose robot simulators such as Stage and ARGoS running on the desktop. Although future work will aim to demonstrate the transferability of the evolved solutions, we note that the fidelity of the simulator is similar to previous work (Jones et al., 2016) which successfully transferred with only moderate reality gap effects.

### 3.3 Image Processing

For the computationally demanding image processing task, Table 6 shows the time taken for the Xpuck to process a 320 × 240 pixel frame using the ArUco library to search for markers. With four large markers, the 23 ms processing time is fast enough to sustain the full camera frame rate of 15 Hz. In the 81 marker case, detection speed slows to 94 ms, such that a 15 Hz rate is not sustainable. In both cases, however, all the markers were correctly detected in each frame.

**Table 5:** Evolutionary algorithm parameters.

Parameter	Value	Description
*n_gens_*	100	Number of generations
*n_sub_*	32	Size of subpopulation
*n_sims_*	8	Number of simulations for evaluation of fitness
*n_elite_*	3	Size of elite
*p_mparam_*	0.05	Probability of mutating a parameter
*p_mnode_*	0.05	Probability of mutating a node
*p_msubtree_*	0.05	Probability of replacing a subtree
*n_depth_*	4	Maximum depth of tree generated
*n_tsize_*	3	Tournament size
*n_epoch_*	2	Migration epoch

**Table 6:** ArUco detector speed at a resolution of 320 × 240 pixels under different conditions.

Condition	Processing time (ms)	*σ*
No markers	12.4	2.7
4 mm × 100 mm markers	23.3	4.5
81 mm × 20 mm markers	93.8	0.25

*In each case, the input was for 60 s. The detector code is unable to process frames at the full 15 Hz in the 81 marker case*.

The dsPIC of the e-puck would not be capable of running this code—it is only capable of capturing camera video at 40 × 40 pixels and 4 Hz with no image processing (Mondada et al., 2009) and has insufficient RAM to hold a complete image. The Linux Extension Board processor could potentially run the detection code, but we estimate the processing time would be at least 50 times longer ^13^ giving a frame rate of less than 1 Hz.

**Figure 7:**
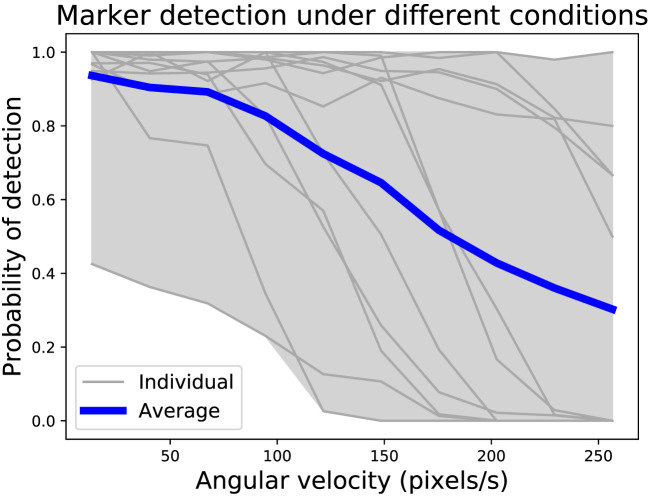
Probability of marker detection under different conditions. There are four markers around the arena, with data collected at three locations, giving twelve distance/angle combinations. Observations at a resolution of 320 × 240 pixels were made for 31,500 frames, with 8,947 marker detections out of a possible 11,076, a detection rate of 81%. The number of detections compared to the maximum possible for each geometry was binned by angular velocity to give probability curves. Gray lines are individual distance/angle combinations, and the blue line is the average over all combinations. Generally, detection rate falls with increasing angular velocity, with a 50% detection rate at 180 pixels/s.

The arena detection experiment collected 31,500 frames, with 11,076 marker detections possible in ideal circumstances. Actual detections numbered 8,947, a total detection rate of 81%. Figure 7 shows the probability of detecting a marker under different conditions. With four markers around the arena, and the Xpuck capturing data at three locations within the arena, there are twelve distance/angle combinations. Distances vary from 0.5 to 1.5 m, and angles from 0° to 70°. The gray envelope and lines show the individual distance/angle combinations against the angular velocity, with the blue line being the average over all observations. Angular velocity is expressed in pixels/s for better intuition about how fast a marker is traversing the field of view of the camera. Generally, the detection rate falls as the angular velocity increases, with a 50% detection rate at 180 pixels/s.

This shows that, even with unoptimized code, the Xpuck has sufficient computational performance, and the camera subsystem is of sufficient quality, that visual marker tracking is feasible.

### 3.4 Evolution

**Figure 8:**
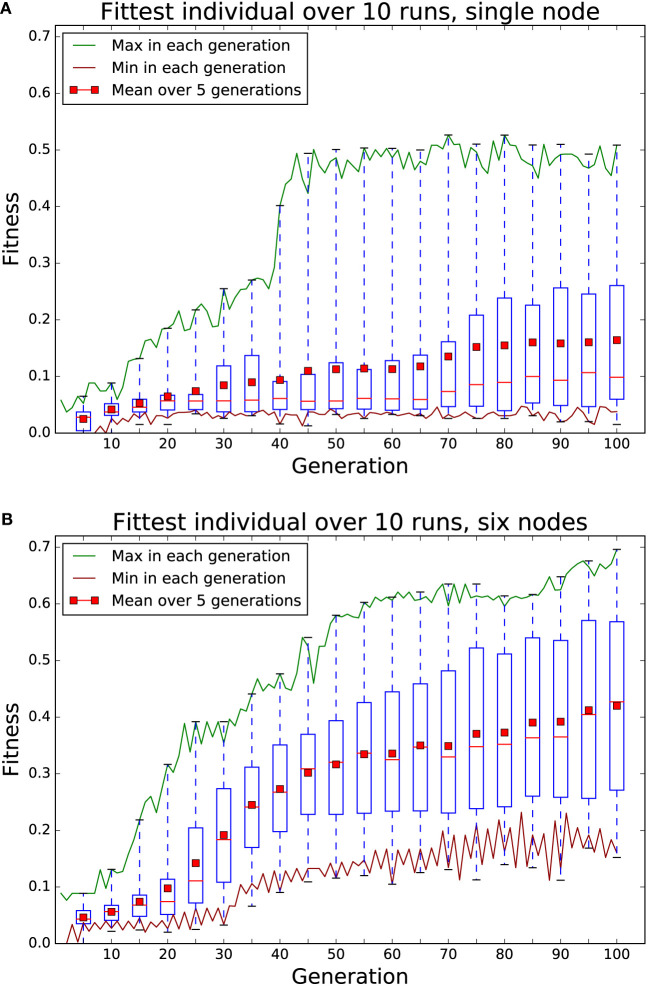
Comparison of 100 generations of evolution using a single node **(A)** and using an island model with six nodes **(B)**. Each node has a population of 32 individuals, evaluated 8 times with different starting conditions for fitness. Each node in the six node system replaces is five least fit individuals with the fittest from the other five nodes every two generations. Boxes summarize data for that generation and the previous four. Red bar in boxes indicates median. The six node system clearly shows higher maximum fitness after 100 generations and reaches the same mean fitness as the single node system in a quarter of the time. The large difference between mean and median in the single node system is due to seven of the ten runs not exceeding a fitness of 0.1.

The results are summarized in Figure 8. It is clear that the six node island model evolutionary system performs better than the single node. Maximum fitness reached is higher at 0.7 vs 0.5, and progress is faster. Of interest is the very low median fitness of the single node populations (shown with red bar in boxes), compared to the mean. This is because seven out of the ten runs never reached a higher fitness than 0.1 suggesting the population size or the number of generations is too small. Conversely, the median and mean of the island model population’s maximum fitnesses are quite similar, showing a more consistent performance across runs. If we look at how fast the mean fitness rises, a single node takes 100 generations for the fitness to reach 0.15. The six node system reaches this level of mean fitness after 25 generation, four times faster.

**Figure 9:**
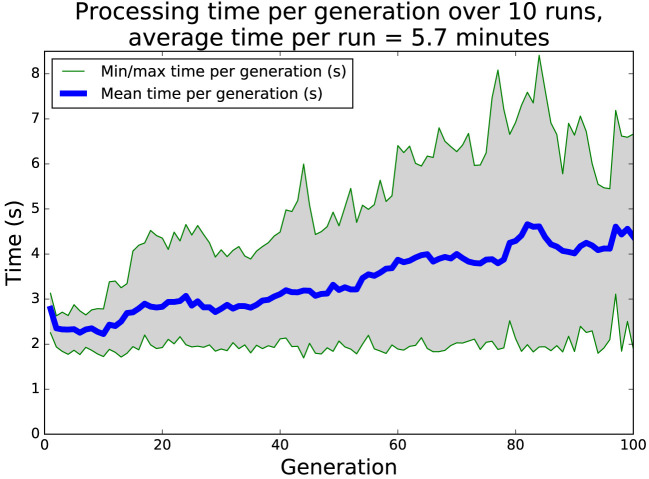
Time per generation of single node evolution, 10 runs of 100 generations each, with different starting conditions. The average length of a run is 5.7 min. The variation in processing time is due mostly to the size and complexity of the behavior trees within the population.

Figure 9 shows a plot of the elapsed processing time per generation over ten runs. The variation is mostly due to the complexity and depth of the behavior tree controllers within each generation, together with the trajectory of the robots in simulation. Each of the ten runs of both the island model and the single node systems completed in less than 10 min. For comparison, each evolutionary run in our previous work (Jones et al., 2016) took several hours on a powerful desktop machine.

This demonstrates the Xpucks are sufficiently capable to host in-swarm evolutionary algorithms that scale in performance with the size of the swarm.

## 4 Discussion

### 4.1 Background and Related Work

In the introduction, we outline three areas which we feel could benefit from the increased processing power of the Xpuck.

Swarm robotics (Sahin, 2005) takes inspiration from collective phenomena in nature, where global behaviors emerge from the local interactions of the agents of the swarm with each other, and with the environment. The design of controllers such that a desired collective behavior emerges is a central problem. Common approaches use bioinspiration, evolution, reverse engineering, and hand-design (Reynolds, 1987; Trianni et al., 2003; Hauert et al., 2009b; Trianni and Nolfi, 2011; Francesca et al., 2014). The controller architectures include neural networks, probabilistic finite state machines, behavior trees, and hybrid combinations (Baldassarre et al., 2003; Francesca et al., 2015; Duarte et al., 2016; Jones et al., 2016). See Francesca and Birattari (2016) for a recent review. When using evolution or other methods of automatic design within an off-line simulated environment, the problem of the transferability of the controller from simulation to real robots arises, the so-called *reality gap*. There are various approaches to alleviating this such as noise injection within a minimal simulation (Jakobi et al., 1995; Jakobi, 1998), making transferability a goal within the evolutionary algorithm (Koos et al., 2013; Mouret and Chatzilygeroudis, 2017), and reducing the representational power of the controller (Francesca et al., 2014, 2015). Embodied evolution directly tests candidate controllers in reality. When applied to swarms (Watson et al., 2002) the evolutionary algorithm is distributed over the robots (Takaya and Arita, 2003; Bredeche et al., 2012; Doncieux et al., 2015). Other approaches use reality sampling to alter the simulated environment to better match true fitnesses (Zagal et al., 2004; O’Dowd et al., 2014). This requires either off-board processing with communication links to the robot or sufficient processing power on the robot to run simulations. Related is the concept of surrogate fitness functions (Jin, 2011) with cheap but inaccurate fitness measures made in simulation and expensive but accurate measures made in reality.

Using internal simulation, models can be means of detecting malfunction and adapting (Bongard et al., 2006), or asking *what-if* questions, so as to evaluate the consequences of possible actions in simulation (Marques and Holland, 2009). This is applied to the fields of both robot safety and machine ethics in Winfield et al. (2014), Winfield (2015), Blum et al. (2018), and Vanderelst and Winfield (2018). It is obvious that any robot relying on simulation for its ethical or safe behavior must embody that simulation and not use unreliable communications links. Swarms are usually assumed to be robust to failure, but Bjerknes and Winfield (2013) show that this is not always the case. By using internal models and observing other agents within the swarm, agents not behaving as predicted can be identified (Millard et al., 2013, 2014).

The social insects that are often the inspiration for swarm robotics are actually far more complex than the commonly used ANN controllers of swarm robot agents. They have many more neurons, and the neurons are behaviorally complex. The computational requirement of simulating biologically plausible neurons can be estimated. The Izhikevich (2003) model is commonly used and reported performances vary between 7 and 50 MFLOPS/neuron (Ananthanarayanan et al., 2009; Fidjeland and Shanahan, 2010; Scorcioni, 2010; Minkovich et al., 2014). The system we describe could plausibly simulate several thousand biologically plausible neurons per Xpuck.

A number of different platforms have been used for swarm robotics research. The e-puck by Mondada et al. (2009) is widely used for experiments with numbers in the tens. Rubenstein et al. (2012) introduced the Kilobot, which enables swarm experiments involving over 1,000 low-cost robots. Both platforms work on a 2D surface. Other platforms include Swarmbots (Dorigo et al., 2004), R-one (McLurkin et al., 2013), and Pheeno (Wilson et al., 2016). Swarm platforms working in 3D are also described, Hauert et al. (2009a) demonstrate Reynolds flocking (Reynolds, 1987) with small fixed-wing drones, see also Kushleyev et al. (2013) and Vásárhelyi et al. (2014). Most described platforms are homogeneous, but heterogeneous examples exist such as the Swarmanoid (Dorigo et al., 2013). Table 1 compares some of these platforms, looking at cost and processing power. It is only with the very recent platforms of the Pi-puck and Pheeno (unavailable at the time of design of Xpuck) that the processing power exceeds 1.2 GFLOPS.

We designed the Xpuck explicitly with the e-puck in mind, because, like many labs, we already have a reasonably large number of them. The e-puck is very successful, with in excess of 3,500 shipped units, perhaps due to its simple reliable design and extendability. Expansion connectors allow additional boards that add capabilities. Three such are relevant here because they extend the processing power of the e-puck. The Linux Extension Board (Liu and Winfield, 2011) adds a 200-MHz Atmel ARM processor running embedded Linux, with WiFi communication. The e-puck extension for Gumstix Overo COM is a board from GCTronic that interfaces a small Linux single board computer, the Gumstic Overo Earthstorm, ^14^ to the e-puck. A recent addition is the Pi-puck (Millard et al., 2017) which provides a means of using the popular Raspberry Pi single board computers to control an e-puck. The extension board connects the Pi to the various interfaces of the e-puck and provides full access to all sensors and actuators except the camera.

### 4.2 Conclusion

We have presented the Xpuck swarm, a new research platform with an aggregate raw processing power in excess of two Teraflops. The swarm of 16 e-puck robots augmented with custom hardware uses the substantial CPU and GPU processing power available from modern mobile System-on-Chip devices; each individual Xpuck has at least an order of magnitude greater compute performance than previous swarm robotics platforms. As well as the robots themselves, we have described the system as a whole that allows us to run new classes of experiments that require high-individual robot computation and large numbers of robots. We foresee many uses such as online evolution or learning of swarm controllers, simulation of what-if questions about possible actions, distributed super-computing for mobile platforms, and real-world applications of swarm robotics that requires image processing, or distributed SLAM.

To demonstrate the capabilities of the system, we have shown the feasibility of running a widely used fiducial marker recognition image processing library, which could form the basis for a distributed swarm localization system. We have implemented a fast robot simulator tailored specifically to run on the GPU of the Xpuck. The performance of this simulator on the Xpuck GPU is comparable to the same code running on the CPU of a much more powerful desktop system, and at least ten times faster than general purpose simulators such as Stage and ARGoS running on the desktop. By using this fast simulator within an island model evolutionary algorithm, we have demonstrated the ability to perform in-swarm evolution. The increasing performance at reaching a given fitness with increasing Xpuck swarm size demonstrates the scalability of this approach. Previous work of ours used evolutionary algorithms that took several hours on the desktop to achieve what is now possible in less than 10 min on the swarm.

In conclusion, we present a new tool for investigating collective behaviors. Our platform provides vastly increased computational performance situated within the swarm itself, opening up the possibility of novel approaches and algorithms.

## Author Contributions

SJ substantial contributions to the conception of the work, design and implementation of robots, design and implementation of software, design of experiments, acquisition and analysis of data, drafting the work, final approval, and agreement to be accountable. MS, SH, and AW substantial contributions to the conception of the work, critical revision of the work, final approval, and agreement to be accountable.

## Conflict of Interest Statement

The authors declare that the research was conducted in the absence of any commercial or financial relationships that could be construed as a potential conflict of interest.

## Footnotes

^1^2004 Nvidia 6800 Ultra 40 GFLOPS 110 W, 0.35 GFLOPS/W. 2014 Samsung Exynos 5422 120 GFLOPS 5 W, 24 GFLOPS/W.

^2^https://developer.arm.com/technologies/big-little.

^3^Essentially, how fast clock frequency will be varied to meet changing CPU load.

^4^https://wiki.linuxfoundation.org/realtime/documentation/howto/tools/cyclictest.

^5^Corruption-proof SD card filesystem for embedded Linux? http://unix.stackexchange.com/questions/136269/corruption-proof-sd-card-filesystem-for-embedded-linux.

^6^or “two-and-a-half D” with sensors having some awareness of Z but kinematics and dynamics modelled purely in 2D.

^7^We achieved 80,000 with our Box2D-based kilobot simulator (Jones et al., 2016).

^8^The six cores are divided into two core groups, one with four cores and one with two. These are presented as two separate OpenCL devices. For ease of coding, only one core group was used.

^9^https://developer.arm.com/products/software-development-tools/ds-5-development-studio/streamline.

^10^Version 1.2, standard install from Ubuntu 14.04.4 ROS Indigo repository.

^11^Online quote from https://www.pcbway.com/.

^12^https://bitbucket.org/siteks/xpuck_design.

^13^ARM926EJS @200 MHz = 220DMIPS, A15 @800 MHz = 2800DMIPS, 4×penalty for no floating point, single core only: 50×.

^14^https://store.gumstix.com/coms/overo-coms/overo-earthstorm-com.html.

## References

[B1] Ananthanarayanan R. Esser S. K. Simon H. D. Modha D. S. (2009). “The cat is out of the bag: cortical simulations with 10^9^ neurons, 10^13^ synapses,” in High Performance Computing Networking, Storage and Analysis, Proceedings of the Conference on (New York, NY: IEEE), 1–12.

[B2] Baldassarre G. Nolfi S. Parisi D. (2003). Evolving mobile robots able to display collective behaviors. Artif. Life 9, 255–267. 10.1162/106454603322392460 14556687

[B3] Bjerknes J. D. Winfield A. F. (2013). “On fault tolerance and scalability of swarm robotic systems,” in Distributed Autonomous Robotic Systems (Berlin: Springer), 431–444.

[B4] Blum C. Winfield A. F. T. Hafner V. V. (2018). Simulation-based internal models for safer robots. Front. Robot. AI 4:74 10.3389/frobt.2017.00074

[B5] Bongard J. Zykov V. Lipson H. (2006). Resilient machines through continuous self-modeling. Science 314, 1118–1121. 10.1126/science.1133687 17110570

[B6] Bredeche N. Montanier J.-M. Liu W. Winfield A. F. (2012). Environment-driven distributed evolutionary adaptation in a population of autonomous robotic agents. Math. Comput. Model. Dyn. Syst. 18, 101–129. 10.1080/13873954.2011.601425

[B7] Catto E. (2009). Box2D: A 2D Physics Engine for Games. Available from: http://box2d.org/about/

[B8] Champandard A. (2007). “Behavior trees for next-gen game AI,” in Game Developers Conference, Audio Lecture, Lyon.

[B9] Colledanchise M. Ogren P. (2014). “How behavior trees modularize robustness and safety in hybrid systems,” in Intelligent Robots and Systems (IROS 2014), 2014 IEEE/RSJ International Conference on (Chicago, IL: IEEE), 1482–1488.

[B10] Doncieux S. Bredeche N. Mouret J.-B. Eiben A. E. G. (2015). Evolutionary robotics: what, why, and where to. Front. Robot. AI 2:4 10.3389/frobt.2015.00004

[B11] Dorigo M. Floreano D. Gambardella L. M. Mondada F. Nolfi S. Baaboura T. (2013). Swarmanoid: a novel concept for the study of heterogeneous robotic swarms. IEEE Robot. Autom. Mag. 20, 60–71. 10.1109/MRA.2013.2252996

[B12] Dorigo M. Tuci E. Groß R. Trianni V. Labella T. H. Nouyan S. (2004). “The swarm-bots project,” in International Workshop on Swarm Robotics (Berlin: Springer), 31–44.

[B13] Duarte M. Gomes J. Costa V. Oliveira S. M. Christensen A. L. (2016). Hybrid Control for a Real Swarm Robotics System in an Intruder Detection Task. Berlin: Springer International Publishing, 213–230.

[B14] Fidjeland A. K. Shanahan M. P. (2010). “Accelerated simulation of spiking neural networks using GPUs,” in Neural Networks (IJCNN), The 2010 International Joint Conference on (Barcelona: IEEE), 1–8.

[B15] Floreano D. Mitri S. Magnenat S. Keller L. (2007). Evolutionary conditions for the emergence of communication in robots. Curr. Biol. 17, 514–519. 10.1016/j.cub.2007.01.058 17320390

[B16] Francesca G. Birattari M. (2016). Automatic design of robot swarms: achievements and challenges. Front. Robot. AI 3:29 10.3389/frobt.2016.00029

[B17] Francesca G. Brambilla M. Brutschy A. Garattoni L. Miletitch R. Podevijn G. (2015). AutoMoDe-chocolate: automatic design of control software for robot swarms. Swarm Intell. 9, 125–152. 10.1007/s11721-015-0107-9

[B18] Francesca G. Brambilla M. Brutschy A. Trianni V. Birattari M. (2014). AutoMoDe: a novel approach to the automatic design of control software for robot swarms. Swarm Intell. 8, 89–112. 10.1007/s11721-014-0092-4

[B19] Garrido-Jurado S. Noz Salinas R. M. Madrid-Cuevas F. Marín-Jiménez M. (2014). Automatic generation and detection of highly reliable fiducial markers under occlusion. Pattern Recognit. 47, 2280–2292. 10.1016/j.patcog.2014.01.005

[B20] Gaul R. (2012). Impulse Engine 2D Physics Simulator. Available from: http://www.randygaul.net/projects-open-sources/impulse-engine/

[B21] Gronqvist J. Lokhmotov A. (2014). “Optimising OpenCL kernels for the ARM Mali-T600 GPUs,” in GPU Pro 5: Advanced Rendering Techniques (Boca Raton, FL: CRC Press), 327–357.

[B22] Gurrum S. P. Edwards D. R. Marchand-Golder T. Akiyama J. Yokoya S. Drouard J.-F. (2012). “Generic thermal analysis for phone and tablet systems,” in Electronic Components and Technology Conference (ECTC), 2012 IEEE 62nd (New York, NY: IEEE), 1488–1492.

[B23] Gutiérrez Á Campo A. Dorigo M. Donate J. Monasterio-Huelin F. Magdalena L. (2009a). “Open e-puck range & bearing miniaturized board for local communication in swarm robotics,” in Robotics and Automation, 2009. ICRA’09. IEEE International Conference on (Kobe: IEEE), 3111–3116.

[B24] Gutiérrez Á Tuci E. Campo A. (2009b). Evolution of neuro-controllers for robots’ alignment using local communication. Int. J. Adv. Robot. Syst. 6, 6 10.5772/6766

[B25] Hauert S. Zufferey J.-C. Floreano D. (2009a). Evolved swarming without positioning information: an application in aerial communication relay. Auton. Robots 26, 21–32. 10.1007/s10514-008-9104-9

[B26] Hauert S. Zufferey J.-C. Floreano D. (2009b). “Reverse-engineering of artificially evolved controllers for swarms of robots,” in Evolutionary Computation, 2009. CEC’09. IEEE Congress on (New York, NY: IEEE), 55–61.

[B27] Hintjens P. (2013). ZeroMQ: Messaging for Many Applications. Sebastopol, CA: O’Reilly Media, Inc.

[B28] Ho J. Smith R. (2015). NVIDIA Tegra X1 Preview and Architecture Analysis. Available at: http://www.anandtech.com/show/8811/nvidia-tegra-x1-preview

[B29] Izhikevich E. M. (2003). Simple model of spiking neurons. IEEE Trans. Neural Networks 14, 1569–1572. 10.1109/TNN.2003.820440 18244602

[B30] Jakobi N. (1998). “Running across the reality gap: Octopod locomotion evolved in a minimal simulation,” in Evolutionary Robotics (Berlin: Springer), 39–58.

[B31] Jakobi N. Husbands P. Harvey I. (1995). “Noise and the reality gap: the use of simulation in evolutionary robotics,” in Advances in Artificial Life (Berlin: Springer), 704–720.

[B32] Jin Y. (2011). Surrogate-assisted evolutionary computation: recent advances and future challenges. Swarm Evol. Comput. 1, 61–70. 10.1016/j.swevo.2011.05.001

[B33] Jones S. Studley M. Hauert S. Winfield A. (2016). “Evolving behaviour trees for swarm robotics,” in Springer Tracts in Advanced Robotics: 13th International Symposium on Distributed Autonomous Robotic Systems (DARS 2016), London, UK.

[B34] Jones S. Studley M. Winfield A. (2015). “Mobile GPGPU acceleration of embodied robot simulation,” in Artificial Life and Intelligent Agents: First International Symposium, ALIA 2014 (Cham: Bangor, UK). Revised Selected Papers, Communications in Computer and Information Science. Springer International Publishing.

[B35] Khronos OpenCL Working Group. (2010). The OpenCL Specification, Version 1.1. Beaverton, OR: Khronos Group Available at: https://www.khronos.org/registry/OpenCL/specs/opencl-1.1.pdf

[B36] Koos S. Mouret J.-B. Doncieux S. (2013). The transferability approach: crossing the reality gap in evolutionary robotics. Evol. Comput. IEEE Trans. 17, 122–145. 10.1109/TEVC.2012.2185849

[B37] Koza J. R. (1992). Genetic Programming: On the Programming of Computers by Means of Natural Selection, Vol. 1 Cambridge, MA: MIT Press.

[B38] Kushleyev A. Mellinger D. Powers C. Kumar V. (2013). Towards a swarm of agile micro quadrotors. Auton. Robot. 35, 287–300. 10.1007/s10514-013-9349-9

[B39] Liu W. Winfield A. F. (2011). Open-hardware e-puck Linux extension board for experimental swarm robotics research. Microprocess. Microsyst. 35, 60–67. 10.1016/j.micpro.2010.08.002

[B40] Mack C. A. (2011). Fifty years of Moore’s law. IEEE Trans. Semicond. Manuf. 24, 202–207. 10.1109/TSM.2010.2096437

[B41] Marques H. G. Holland O. (2009). Architectures for functional imagination. Neurocomputing 72, 743–759. 10.1016/j.neucom.2008.06.016

[B42] McLurkin J. Lynch A. J. Rixner S. Barr T. W. Chou A. Foster K. (2013). “A low-cost multi-robot system for research, teaching, and outreach,” in Distributed Autonomous Robotic Systems (Berlin: Springer), 597–609.

[B43] Menzel R. Giurfa M. (2001). Cognitive architecture of a mini-brain: the honeybee. Trends Cogn. Sci. 5, 62–71. 10.1016/S1364-6613(00)01601-6 11166636

[B44] Millard A. G. Joyce R. A. Hilder J. A. Fleseriu C. Newbrook L. Li W. (2017). “The Pi-puck extension board: a Raspberry Pi interface for the e-puck robot platform,” in IEEE/RSJ International Conference on Intelligent Robots and Systems (York, UK: York).

[B45] Millard A. G. Timmis J. Winfield A. F. (2013). “Towards exogenous fault detection in swarm robotic systems,” in Conference towards Autonomous Robotic Systems (Berlin: Springer), 429–430.

[B46] Millard A. G. Timmis J. Winfield A. F. (2014). “Run-time detection of faults in autonomous mobile robots based on the comparison of simulated and real robot behaviour,” in Intelligent Robots and Systems (IROS 2014), 2014 IEEE/RSJ International Conference on (Chicago, IL: IEEE), 3720–3725.

[B47] Minkovich K. Thibeault C. M. O’Brien M. J. Nogin A. Cho Y. Srinivasa N. (2014). HRLSim: a high performance spiking neural network simulator for GPGPU clusters. IEEE Trans. Neural Networks Learn. Syst. 25, 316–331. 10.1109/TNNLS.2013.2276056 24807031

[B48] Mitri S. Floreano D. Keller L. (2009). The evolution of information suppression in communicating robots with conflicting interests. Proc. Natl. Acad. Sci. U.S.A. 106, 15786–15790. 10.1073/pnas.0903152106 19805224 PMC2747196

[B49] Mondada F. Bonani M. Raemy X. Pugh J. Cianci C. Klaptocz A. (2009). “The e-puck, a robot designed for education in engineering,” in Proceedings of the 9th Conference on Autonomous Robot Systems and Competitions, Castelo Branco, Vol. 1, 59–65.

[B50] Mouret J.-B. Chatzilygeroudis K. (2017). “20 years of reality gap: a few thoughts about simulators in evolutionary robotics,” in Workshop “Simulation in Evolutionary Robotics”, Genetic and Evolutionary Computation Conference, New York, NY.

[B51] Niiranen J. (1999). “Fast and accurate symmetric Euler algorithm for electromechanical simulations NOTE: the method became later known as “Symplectic Euler”,” in Proceedings of the 6th International Conference ELECTRIMACS ’99: Modelling and Simulation of Electric Machines, Converters and Systems, Vol. 1 (Lisbon: Lisboa, Portugal), 71–78.

[B52] Nvidia. (2007). NVIDIA CUDA, Compute Unified Device Architecture Programming Guide. Santa Clara, CA: NVIDIA.

[B53] O’Dowd P. J. Studley M. Winfield A. F. (2014). The distributed co-evolution of an on-board simulator and controller for swarm robot behaviours. Evol. Intell. 7, 95–106. 10.1007/s12065-014-0112-8

[B54] Ogren P. (2012). “Increasing modularity of UAV control systems using computer game behavior trees,” in AIAA Guidance, Navigation and Control Conference (Minneapolis, MN).

[B55] Pinciroli C. Trianni V. O’Grady R. Pini G. Brutschy A. Brambilla M. (2011). “ARGoS: a modular, multi-engine simulator for heterogeneous swarm robotics,” in Intelligent Robots and Systems (IROS), 2011 IEEE/RSJ International Conference on (San Francisco, CA: IEEE), 5027–5034.

[B56] Poli R. Langdon W. B. McPhee N. F. Koza J. R. (2008). A Field Guide to Genetic Programming. Available at: http://www.gp-field-guide.org.uk

[B57] Polilov A. A. (2012). The smallest insects evolve anucleate neurons. Arthropod Struct. Dev. 41, 29–34. 10.1016/j.asd.2011.09.001 22078364

[B58] Quigley M. Conley K. Gerkey B. Faust J. Foote T. Leibs J. (2009). “ROS: an open-source robot operating system,” in ICRA Workshop on Open Source Software, Vol. 3 (Kobe, Japan), 5.

[B59] Reynolds C. W. (1987). “Flocks, herds and schools: a distributed behavioral model,” in ACM SIGGRAPH Computer Graphics (New York, NY: ACM), 21, 25–34.

[B60] Rodeh O. Bacik J. Mason C. (2013). BTRFS: the Linux B-tree filesystem. ACM Trans. Storage 9, 9 10.1145/2501620.2501623

[B61] Rostedt S. Hart D. V. (2007). “Internals of the RT patch,” in Proceedings of the Linux Symposium, Ottawa, Vol. 2, 161–172.

[B62] Rubenstein M. Ahler C. Nagpal R. (2012). “Kilobot: a low cost scalable robot system for collective behaviors,” in Robotics and Automation (ICRA), 2012 IEEE International Conference on (St Paul, MA: IEEE), 3293–3298.

[B63] Sahin E. (2005). “Swarm robotics: from sources of inspiration to domains of application,” in Swarm Robotics (Berlin: Springer), 10–20.

[B64] Scheper K. Y. Tijmons S. de Visser C. C. de Croon G. C. (2016). Behavior trees for evolutionary robotics. Artif. Life. 22, 23–48. 10.1162/ARTL_a_00192 26606468

[B65] Scorcioni R. (2010). “GPGPU implementation of a synaptically optimized, anatomically accurate spiking network simulator,” in Biomedical Sciences and Engineering Conference (BSEC), 2010 (Oak Ridge, TN: IEEE), 1–3.

[B66] Takaya Y. U. Arita T. (2003). “Situated and embodied evolution in collective evolutionary robotics,” in Proc. of the 8th International Symposium on Artificial Life and Robotics (Beppu: Citeseer).

[B67] Thrun S. Burgard W. Fox D. (2005). Probabilistic Robotics. Intelligent Robotics and Autonomous Agents. Cambridge, MA: MIT Press.

[B68] Trianni V. Groß R. Labella T. H. Sahin E. Dorigo M. (2003). “Evolving aggregation behaviors in a swarm of robots,” in Advances in Artificial Life (Berlin: Springer), 865–874.

[B69] Trianni V. Nolfi S. (2011). Engineering the evolution of self-organizing behaviors in swarm robotics: a case study. Artif. Life 17, 183–202. 10.1162/artl_a_00031 21554112

[B70] Vanderelst D. Winfield A. (2018). An architecture for ethical robots inspired by the simulation theory of cognition. Cogn. Syst. Res. 48, 56–66. 10.1016/j.cogsys.2017.04.002

[B71] Vásárhelyi G. Virágh C. Somorjai G. Tarcai N. Szörényi T. Nepusz T. (2014). “Outdoor flocking and formation flight with autonomous aerial robots,” in Intelligent Robots and Systems (IROS 2014), 2014 IEEE/RSJ International Conference on (Chicago, IL: IEEE), 3866–3873.

[B72] Vaughan R. (2008). Massively multi-robot simulation in stage. Swarm Intell. 2, 189–208. 10.1007/s11721-008-0014-4

[B73] Watson R. A. Ficici S. G. Pollack J. B. (2002). Embodied evolution: distributing an evolutionary algorithm in a population of robots. Rob. Auton. Syst. 39, 1–18. 10.1016/S0921-8890(02)00170-7

[B74] White J. G. Southgate E. Thomson J. N. Brenner S. (1986). The structure of the nervous system of the nematode *Caenorhabditis elegans*. Philos. Trans. R. Soc. Lond. B Biol. Sci. 314, 1–340. 10.1098/rstb.1986.0056 22462104

[B75] Whitley D. Rana S. Heckendorn R. B. (1999). The island model genetic algorithm: on separability, population size and convergence. CIT J. Comput. Inform. Technol. 7, 33–47.

[B76] Williams R. L. Carter B. E. Gallina P. Rosati G. (2002). Dynamic model with slip for wheeled omnidirectional robots. IEEE Trans. Robot. Autom. 18, 285–293. 10.1109/TRA.2002.1019459

[B77] Wilson S. Gameros R. Sheely M. Lin M. Dover K. Gevorkyan R. (2016). Pheeno, a versatile swarm robotic research and education platform. IEEE Robot. Autom. Lett. 1, 884–891. 10.1109/LRA.2016.2524987

[B78] Winfield A. F. (2015). “Robots with internal models: a route to self-aware and hence safer robots,” in The Computer after Me: Awareness and Self-Awareness in Autonomic Systems (London, UK: World Scientific), 237–252.

[B79] Winfield A. F. Blum C. Liu W. (2014). “Towards an ethical robot: internal models, consequences and ethical action selection,” in TAROS 2014 – Towards Autonomous Robotic Systems, volume 8717 of Lecture Notes in Computer Science, eds Mistry M. Leonardis A. Witkowski M. Melhuish C. (Berlin: Springer International Publishing), 85–96.

[B80] Xianyi Z. Qian W. Yunquan Z. (2012). “Model-driven level 3 BLAS performance optimization on Loongson 3A processor,” in Parallel and Distributed Systems (ICPADS), 2012 IEEE 18th International Conference on (Singapore: IEEE), 684–691.

[B81] Zagal J. C. Ruiz-del Solar J. Vallejos P. (2004). “Back to reality: crossing the reality gap in evolutionary robotics,” in IAV 2004 the 5th IFAC Symposium on Intelligent Autonomous Vehicles (Lisbon, Portugal).

